# The association between leptin, interleukin-6, and hip radiographic osteoarthritis in older people: a cross-sectional study

**DOI:** 10.1186/ar3022

**Published:** 2010-05-19

**Authors:** Oliver P Stannus, Graeme Jones, Stephen J Quinn, Flavia M Cicuttini, Dawn Dore, Changhai Ding

**Affiliations:** 1 Menzies Research Institute, University of Tasmania, Private Bag 23, Hobart, Tasmania 7000, Australia; 2 Department of Epidemiology and Preventive Medicine, Monash University, 89 Commercial Road, Melbourne 3004, Australia

## Abstract

**Introduction:**

The associations between leptin, interleukin (IL)-6, and hip radiographic osteoarthritis (OA) have not been reported, and their roles in obesity-related hip OA are unclear. The aim of this study was to describe the associations between leptin, IL-6, and hip radiographic osteoarthritis (ROA) in older adults.

**Methods:**

A cross-sectional sample of 193 randomly selected subjects (mean age, 63 years; range, 52 to 78 years; 48% female subjects) were studied. Hip ROA, including joint-space narrowing (JSN) and osteophytes, was determined by anteroposterior radiograph. Serum levels of leptin and interleukin (IL)-6 were measured with radioimmunoassay. Fat mass was measured with dual-energy x-ray absorptiometry (DXA). Body mass index (BMI) and waist-to-hip ratio (WHR) were calculated.

**Results:**

In multivariable analysis, hip JSN was associated with serum levels of leptin in the whole sample (β = 0.046 per μg/L, *P *= 0.024 for superior; β = 0.068 per μg/L, *P *= 0.004 for axial compartment) and IL-6 only in females (β = 0.241 per pg/ml, *P *= 0.002 for superior; β = 0.239 per pg/ml, *P *= 0.001 for axial compartment). The positive associations between body-composition measures (BMI, WHR, percentage total fat mass, and percentage trunk fat mass) and hip JSN in women became nonsignificant after adjustment for leptin but not for IL-6. No significant associations were found between leptin, IL-6, and the presence or severity of osteophytes.

**Conclusions:**

This study suggests that metabolic and inflammatory mechanisms may play a role in the etiology of hip OA and that the associations between body composition and hip JSN are mediated by leptin, particularly in women.

## Introduction

Osteoarthritis (OA) is a multifactorial disease of the joints characterized by gradual loss of articular cartilage. The main risk factors for OA are age [1], female sex [2], and obesity [1]. Studies of hip OA among predominantly white populations have estimated prevalence rates of ~5-7%, which are higher in women [3,4]. Body mass index (BMI) is strongly associated with prevalence [5] and incidence [6-8] of knee OA. Although inconsistent associations have been reported between BMI and hip OA [7,9-11], a systematic review has suggested that BMI is moderately associated with hip OA [12].

Metabolic changes associated with obesity are a possible causative pathway for OA [13]. Leptin is a 16-kDa protein encoded by the gene obese (ob) to regulate food intake and energy expenditure and is correlated with BMI and female sex [14]. It is secreted mainly by adipocytes [15], but also by chondrocytes [16,17], and its production is increased in the cartilage of OA subjects [16]. Leptin levels in synovial fluid are correlated with BMI [16]; thus, it is a possible metabolic factor in OA pathogenesis [18], appearing to mediate obesity- and sex-related knee cartilage loss [14]. Leptin is now regarded as a proinflammatory adipocytokine [19] that belongs structurally to the interleukin (IL)-6 family of cytokines [20,21]. The exact function of leptin in OA is undetermined, although it is proposed to have a biphasic effect [22], with low levels facilitating cartilage synthesis, and excess leptin causing cartilage inflammation and degeneration [23]. Although the prevalence of hip OA is lower than that of knee OA, and some risk factors such as obesity appear not to have equal effects on hip and knee OA, effects of leptin on hip and knee OA may be different. However, as far as we are aware, the associations between leptin and hip OA have not been reported.

IL-6 is a cytokine with pro- and antiinflammatory effects, produced by various types of lymphoid and nonlymphoid cells as well as chondrocytes and osteoblasts [24,25]. Like its cousin leptin, IL-6 can be expressed by adipose tissue [26] and may have mixed roles in OA; IL-6 is able to downregulate catabolic factors involved in cartilage degeneration [27,28], but can itself induce inflammation. Increased IL-6 expression has been observed in subchondral bone and osteophytes of subjects with knee OA [25,29]. Circulating levels of both IL-6 and leptin have been associated with knee OA [14,30]. The functions of IL-6 in hip OA are unclear, and whether it is involved in any systemic or leptin-mediated process is unknown. The aim of this study was, therefore, to describe the relations between radiographic hip OA and circulating levels of leptin and IL-6 among older adults.

## Materials and methods

### Subjects

Subjects between ages 50 and 79 years were selected randomly from the roll of electors in southern Tasmania (population, 229,000) with an equal number of men and women. Institutionalized persons were excluded. This study was conducted as part of the Tasmanian Older Adult Cohort Study (TASOAC), an ongoing, prospective, population-based study in 1,100 subjects aimed at identifying the environmental, genetic, and biochemical factors associated with the development and progression of osteoarthritis and osteoporosis (the overall response rate was 57%). The study was approved by the Southern Tasmanian Health and Medical Human Research Ethics Committee, and written informed consent was obtained from all participants. We selected the first 193 subjects to perform serum measurements. Self-report of smoking status and diseases including rheumatoid arthritis, asthma, cardiovascular disease, and diabetes were recorded by questionnaire. Hip pain was assessed by questionnaire and was defined as pain for >24 hours in the last 12 months or daily pain on >30 days of the last year.

### Anthropometrics

Height was measured to the nearest 0.1 cm (with shoes, socks, and headgear removed) by using a stadiometer. Weight was measured to the nearest 0.1 kg (with shoes, socks, and bulky clothing removed) by using a single pair of electronic scales (Seca Delta Model 707, Bradford, MA) that were calibrated by using a known weight at the beginning of each clinic. Body mass index (BMI; weight (kg)/height^2 ^(m^2^)) was also calculated. Waist and hip circumference were measured and waist-to-hip ratio (WHR; waist circumference (m)/hip circumference (m)) was calculated.

Total body and trunk fat mass (kg) was measured by a Hologic dual energy x-ray absorptiometry (DXA) scanner (Hologic Corp., Waltham, MA, USA). Percentage total body or trunk fat mass is the ratio of total body or trunk fat mass divided by total body or trunk mass (*i.e*., the sum of fat mass, lean mass, and bone mass). Nontrunk fat mass (kg) was calculated by subtracting trunk fat mass from total body fat mass.

### Serum measurement

Serum was isolated and refrigerated overnight in plastic tubes, at which time, aliquots were prepared and stored at -80°C. The levels of total leptin were measured with radioimmunoassay (LINCO Research; now part of Millipore, MO, USA) with a specificity of 100%. IL-6 was measured with a solid-phase, two-site chemiluminescent enzyme immunometric assay method by use of Immulite IL-6 (EURO/DPC Llanberis, Gwynedd, UK). Samples with undetectable concentrations were assigned a value corresponding to the lower limit of detection of the assay (sensitivities: 0.5 μg/L for leptin and 2 pg/ml for IL-6). The coefficients of variation (CVs) in our hands were ~5% for leptin and 8% for IL-6, as previously published [14,31].

### Radiographic measurements

Anteroposterior radiographs of the pelvis with weight bearing and with both feet in 10 degrees of internal rotation were obtained. Radiographic features of joint-space narrowing (JSN) (superior and axial) and osteophytes (superior femoral and superior acetabular) of the left and right hip were graded on a 4-point scale (range, 0 to 3, where 0 = no disease and 3 = most-severe disease) by using the Altman atlas [32], as previously described [33]. Each score was arrived at by consensus between two readers who simultaneously assessed the radiograph, with immediate reference to the atlas. The intraobserver reliability was assessed in 40 subjects, with intraclass correlation coefficients of 0.60 to 0.87 [33].

### Data analysis

Student *t *or Mann-Whitney *U *tests (where appropriate) were used to compare means or proportions. Bar graphs were used to depict median leptin or IL-6 levels for hips grouped by grade of severity in either compartment.

Because of the low prevalence of grade 3 JSN in this sample, we combined grades 2 and 3 for analyses. Univariable and multivariable ordered logistic (ordinal) regressions with a partial proportional odds model were used to examine the associations between JSN and leptin or IL-6 before and after adjustment for age, sex, BMI, ever smoking, and disease status (diabetes, rheumatoid arthritis, cardiovascular disease, and asthma) in the whole sample, as well as in men or women separately. These associations were further tested by adjusting for IL-6 for leptin or leptin for IL-6. In this case, ordinal regression provides, for every unit increase of predictor variable, a ratio of the odds of JSN severity being greater than or equal to *k *versus less than *k*, where *k *may be 1 or 2. For ordered variables, this proportional odds ratio is assumed equal over all values of *k*. In the case of a predictor failing to meet this assumption, a partial proportional odds model provides a separate odds ratio for each *k*. β coefficients were given by the natural logarithms of the odds ratios. The associations between JSN and body-composition measures (BMI, WHR, trunk fat ratio, and total fat ratio) were also investigated by ordinal regression with adjustment for leptin or IL-6. A *P *value of < 0.05 (two-tailed) or a 95% confidence interval not including the null point were considered statistically significant. All statistical analyses were performed on Stata V.10.1 for Windows (StataCorp, College Station, TX, USA), and ordinal regressions were performed by using Williams' *gologit2 *function for Stata [34].

## Results

A total of 193 subjects (48% women, aged 52 to 78 years; mean, 63 years) participated in the present study. Radiographs were unreadable in 12 subjects, and leptin was not measured in three others because of insufficient serum samples. Two IL-6 measurements were excluded as outliers (>30 pg/ml). No significant differences were found in demographic factors between those selected for serum measurement for this study and the remaining TASOAC cohort for age, female sex, and BMI (data not shown). There were 53% subjects having either a JSN or osteophyte score of ≥ 1 in the hip. Characteristics of the subjects are presented (Table 1). No significant differences were noted between men and women in terms of age, BMI, prevalent rheumatoid arthritis and diabetes, osteophytes, and IL-6 levels; however, women had significantly lower lean mass and waist-to-hip ratio, higher percentage total fat mass and trunk fat mass, and had higher levels of leptin. Women had a higher prevalence of axial JSN (grade >0) and asthma, and a lower prevalence of past smoking, cardiovascular diseases, and hip pain.

**Table 1:** Characteristics of participants

	Both sexes	Males	Females	*P*
	n = 193	n = 100	n = 93	
Age, years	62.6 (7.1)	63.3 (7.2)	61.9 (6.9)	0.168^a^
Body mass index, kg/m^2^	27.6 (4.4)	27.2 (3.9)	28.0 (4.9)	0.230^a^
Total lean mass, kg	52.7 (10.8)	**60.3 (9.1)**	**44.6 (5.1)**	**<0.001^a^**
Total fat mass, %	33.7 (7.6)	**27.5 (3.8)**	**40.3 (4.6)**	**<0.001^a^**
Trunk fat mass, %	33.1 (7.6)	**28.5 (5.5)**	**38.0 (6.3)**	**<0.001^a^**
Waist/hip ratio, %	93.4 (8.1)	**98.6 (5.4)**	**87.7 (6.6)**	**<0.001^a^**
Ever smoked, %	51	**62**	**40**	**0.002**
Rheumatoid arthritis, %	11	8	14	0.183
Asthma, %	15	**9**	**21**	**0.024**
Cardiovascular diseases, %	4	**7**	**1**	**0.039**
Diabetes, %	6	3	9	0.093
Hip pain, %	54	**63**	**45**	**0.013**
Superior joint-space narrowing, %	41	38	45	0.286
Axial joint-space narrowing, %	30	**22**	**40**	**0.008**
Any joint-space narrowing, %	48	43	52	0.212
Any osteophytes, %	25	28	23	0.419
Leptin, pg/ml	13.0 (11.6)	**6.6 (5.6)**	**19.8 (12.5)**	**<0.001^a^**
IL-6, μg/L	3.5 (1.9)	3.5 (1.9)	3.4 (2.0)	0.672^a^

Data shown are mean (SD), except for prevalence rates. ^a^Significance of sex difference by the Student t test, otherwise Mann-Whitney U test. IL-6, interleukin-6.

A significant positive association between IL-6 and leptin was found after adjustment for age, sex, BMI, smoking history, and disease status (*r *= 0.16; *P *= 0.032), although this association became nonsignificant when either sex group was analyzed.

**Figure 1:**
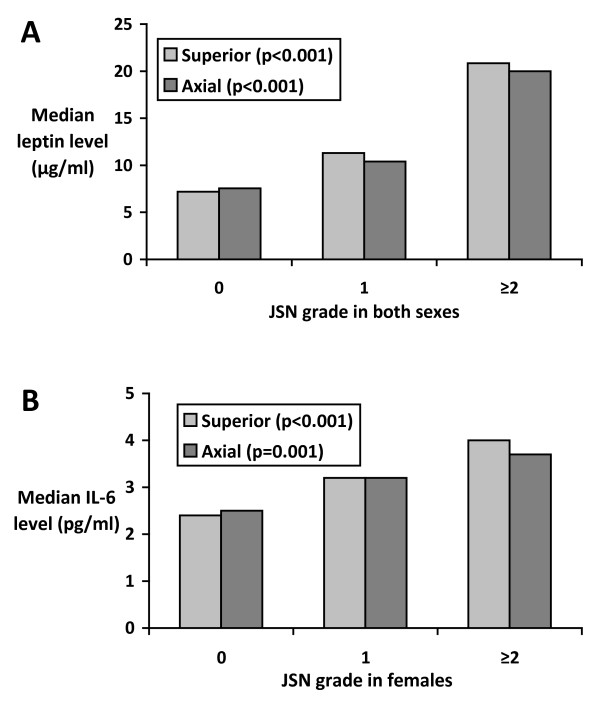
**Associations between hip-joint-space narrowing (JSN)**. **(a) **Leptin in both sexes. **(b) **Interleukin (IL)-6 in women only. Statistical significance calculated by univariable ordinal regression.

Increasing leptin levels were significantly associated with severity of hip JSN for either compartment in unadjusted analyses (superior and axial, both *P *< 0.001) (Figure 1a, Table 2). After adjustment for age, sex, BMI, smoking history, and disease status, serum leptin levels were significantly positively associated with hip JSN in both compartments, for separate and combined sex groups, with the exception of superior JSN in males (Table 2). To illustrate, for instance, among women, an increase of 10 μg/L of leptin was associated with an increase of 10 × 0.043 = 0.43 in superior JSN grade. After inclusion of IL-6 in the model, the existing associations were weaker and became of borderline statistical significance, except in the axial compartment for women and both sexes combined.

**Table 2:** Associations between hip JSN and serum leptin or IL-6 levels

	Univariable		Multivariable^a^		Multivariable^b^	
			
	β (CI)	*P*	β (CI)	*P*	β (CI)	*P*
Leptin (grade per μg/L)						
superior JSN						
Male	0.063 (-0.005 to 0.131)	0.070	0.060 (-0.012 to 0.132)	0.100	0.056 (-0.019 to 0.132)	0.145
Female	**0.046 (0.021 to 0.071)**	**<0.001**	**0.043 (0.004 to 0.083)**	**0.030**	0.038 (-0.001 to 0.078)	0.057
Both sexes	**0.047 (0.026 to 0.069)**	**<0.001**	**0.046 (0.006 to 0.086)**	**0.024**	0.041 (0.000 to 0.082)	0.051
Axial JSN						
Male	0.051 (-0.024 to 0.126)	0.182	**0.081 (0.007 to 0.154)**	**0.031**	0.077 (-0.002 to 0.155)	0.055
Female	**0.041 (0.016 to 0.066)**	**0.002**	**0.066 (0.017 to 0.115)**	**0.008**	**0.060 (0.011 to 0.109)**	**0.017**
Both sexes	**0.052 (0.029 to 0.074)**	**<0.001**	**0.068 (0.022 to 0.115)**	**0.004**	**0.063 (0.014 to 0.112)**	**0.012**
IL-6 (grade per pg/ml)						
Superior JSN						
Male	-0.055 (-0.260 to 0.151)	0.603	-0.066 (-0.268 to 0.136)	0.524	-0.062 (-0.270 to 0.146)	0.556
Female	**0.257 (0.115 to 0.400)**	**<0.001**	**0.241 (0.087 to 0.394)**	**0.002**	**0.202 (0.044 to 0.360)**	**0.012**
Both sexes	0.124 (-0.020 to 0.267)	0.092	0.101 (-0.042 to 0.243)	0.166	0.079 (-0.070 to 0.228)	0.297
Axial JSN						
Male	-0.136 (-0.420 to 0.148)	0.349	-0.138 (-0.409 to 0.134)	0.321	-0.166 (-0.418 to 0.086)	0.197
Female	**0.212 (0.085 to 0.338)**	**0.001**	**0.239 (0.101 to 0.377)**	**0.001**	**0.199 (0.057 to 0.341)**	**0.006**
Both sexes	0.103 (-0.025 to 0.230)	0.116	0.112 (-0.010 to 0.233)	0.071	0.073 (-0.056 to 0.201)	0.267

^a^Adjusted for age, sex if both sexes, BMI, ever smoked, diabetes, rheumatoid arthritis, heart disease, and asthma. ^b^Further adjusted for leptin if IL-6, or IL-6 if leptin. Dependent variable, JSN grade (0-2) in superior or axial compartment. Independent variable, serum level of leptin (μg/L) or IL-6 (pg/ml). IL-6, interleukin-6; CI, confidence interval; JSN, joint-space narrowing; BMI, body mass index.

The interaction term for sex and IL-6 on JSN was significant (*P *< 0.01), so the analyses of associations between IL-6 and JSN were performed separately for women and men. In women, serum IL-6 was significantly associated with JSN (superior and axial, both *P *≤ 0.001) (Figure 1b, Table 2), and in men, no significant associations were seen between IL-6 and JSN (Table 2). After adjustment for the covariates, IL-6 levels were significantly positively associated with JSN in either compartment among women (Table 2). These associations decreased by 16% to 17% but remained significant after adjustment for leptin.

BMI, WHR, total fat ratio, and trunk-fat ratio were each significantly associated with leptin (all *P *< 0.001) after adjustment for age, sex, height, ever smoked, diabetes, rheumatoid arthritis, cardiovascular disease, and asthma (data not shown), whereas only total fat and trunk-fat ratios were also associated with IL-6 (both *P *< 0.05). All four of these anthropometric measures were associated with superior and/or axial JSN in women (Table 3), whereas none was associated with JSN in men (Table 4). For instance, among women, an increase of 5 kg/m^2 ^of BMI was associated with an increase of 5 × 0.11 = 0.55 in superior JSN grade, and an increase of 10% in WHR was associated with an increase of 0.10 × 8.77 = 0.88 in axial JSN grade. All associations between anthropometric measures and JSN among women decreased substantially in magnitude and became statistically nonsignificant after adjustment for leptin, but remained largely unchanged after adjustment for IL-6 (Table 3). Nontrunk fat mass was not associated with hip JSN in either group (data not shown).

**Table 3:** Associations between body composition and hip JSN in women

	Multivariable^a^		Multivariable^b^		Multivariable^c^	
			
	β (CI)	*P*	β (CI)	*P*	β (CI)	*P*
Superior JSN						
BMI (grade per kg/m^2^)	**0.11 (0.03 to 0.19)**	**0.009**	-0.01 (-0.16 to 0.14)	0.874	**0.11 (0.03 to 0.19)**	**0.009**
WHR (grade per 100%)	6.86 (-0.36 to 14.07)	0.063	2.69 (-5.05 to 10.42)	0.496	6.04 (-1.05 to 13.14)	0.095
Trunk fat (grade per 100%)	**12.61 (5.35 to 19.87)**	**0.001**	7.06 (-3.57 to 17.69)	0.193	**11.80 (4.53 to 19.07)**	**0.001**
Total fat (grade per 100%)	**13.17 (3.65 to 22.70)**	**0.007**	3.40 (-11.19 to 17.99)	0.648	**12.10 (2.60 to 21.60)**	**0.013**
Axial JSN						
BMI (grade per kg/m^2^)	0.08 (-0.01 to 0.16)	0.071	-0.12 (-0.28 to 0.05)	0.174	0.07 (-0.01 to 0.16)	0.084
WHR (grade per 100%)	**8.77 (1.39 to 16.14)**	**0.020**	4.88 (-2.88 to 12.64)	0.218	**7.58 (0.55 to 14.62)**	**0.034**
Trunk fat (grade per 100%)	**8.10 (1.53 to 14.66)**	**0.016**	-0.65 (-10.42 to 9.11)	0.896	**6.66 (0.03 to 13.29)**	**0.049**
Total fat (grade per 100%)	7.93 (-0.62 to 16.48)	0.069	-7.18 (-19.50 to 5.14)	0.253	5.64 (-2.79 to 14.08)	0.190

^a^Adjusted for age, height, ever smoked, diabetes, rheumatoid arthritis, heart disease, and asthma. ^b^Further adjusted for leptin or ^c^further adjusted for interleukin-6. Dependent variable, JSN grade (0-2) in superior or axial compartment. Independent variable, BMI, WHR, trunk fat, or total fat (all except BMI expressed as percentages between 0 and 100%). IL-6, interleukin-6; CI, confidence interval; JSN, joint-space narrowing; BMI, body mass index; WHR, waist-to-hip ratio.

**Table 4:** Associations between body composition and hip JSN in men

	Multivariable^a^		Multivariable^b^		Multivariable^c^	
			
	β (CI)	*P*	β (CI)	*P*	β (CI)	*P*
Superior JSN						
BMI (grade per kg/m^2^)	0.10 (0.00 to 0.21)	0.061	0.06 (-0.04 to 0.17)	0.242	0.10 (0.00 to 0.20)	0.058
WHR (grade per 100%)	1.99 (-5.49 to 9.48)	0.602	0.64 (-6.52 to 7.80)	0.861	1.82 (-5.68 to 9.31)	0.635
Trunk fat (grade per 100%)	2.40 (-6.75 to 11.56)	0.607	1.31 (-7.52 to 10.14)	0.771	2.42 (-6.86 to 11.70)	0.609
Total fat (grade per 100%)	2.32 (-10.03 to 14.67)	0.713	-0.12 (-11.69 to 11.45)	0.983	2.30 (-10.24 to 14.84)	0.719
Axial JSN						
BMI (grade per kg/m^2^)	0.09 (-0.04 to 0.22)	0.165	0.02 (-0.09 to 0.13)	0.750	0.09 (-0.03 to 0.21)	0.150
WHR (grade per 100%)	2.29 (-7.66 to 12.25)	0.652	-0.41 (9.77 to 8.96)	0.932	1.94 (-7.88 to 11.75)	0.699
Trunk fat (grade per 100%)	0.08 (-11.25 to 11.42)	0.988	-1.87 (-11.26 to 7.52)	0.697	-0.41 (-12.19 to 11.37)	0.945
Total fat (grade per 100%)	-3.45 (-18.94 to 12.04)	0.662	-6.16 (-18.76 to 6.45)	0.338	-4.25 (-20.71 to 12.22)	0.613

^a^Adjusted for age, height, ever smoked, diabetes, rheumatoid arthritis, heart disease, and asthma. ^b^Further adjusted for leptin or ^c^further adjusted for interleukin-6. Dependent variable, JSN grade (0-2) in superior or axial compartment. Independent variable, BMI, WHR, trunk fat, or total fat (all except BMI expressed as percentages between 0 and 100%). IL-6, interleukin-6; CI, confidence interval; JSN, joint-space narrowing; BMI, body mass index; WHR, waist-to-hip ratio.

Similar results were obtained for these analyses after exclusion of subjects with rheumatoid arthritis (data not shown). In multivariable analysis, hip pain was not significantly associated with either leptin or IL-6 (data not shown). We found no significant relations between leptin, IL-6, and the presence or severity of osteophytes (data not shown).

## Discussion

This cross-sectional study is the first, to our knowledge, to describe the associations between leptin, IL-6, body composition, and hip OA in older adults. We found that serum leptin levels in both sexes and serum IL-6 levels in women were positively associated with hip JSN, but not with osteophytes. The associations were in part dependent on each other, but independent of potential confounders, including age, sex, and BMI. Furthermore, we found that the associations in women between hip JSN and BMI, fat mass, and WHR were dependent on serum leptin levels, but not on serum IL-6 levels.

Emerging evidence suggests that leptin may play a role in knee OA. Chondrocytes possess functional leptin receptors, and low levels of leptin can promote chondrocyte proliferation [35,36] and proteoglycan synthesis [37]. However, overproduction of leptin can increase chondrocyte production of IL-1β and matrix metalloproteinases (MMPs) [23], and in conjunction with IL-1 or interferon (IFN)-γ, induce nitric oxide synthase (NOS) [38,39], which accelerates cartilage degradation. Leptin can be produced within the knee joint [16,40], and concentrations of leptin in synovial fluid have been observed to be similar to or even higher than those in serum [23,40], suggesting leptin may have a local influence on the joint. However, leptin may also contribute to cartilage destruction through a systemic effect, as suggested by our previous study, negatively associating serum leptin levels with knee cartilage volume [14], and as well as studies showing that BMI was associated with leptin concentrations in synovial fluid [16], and that leptin-deficient mice were less likely to have obesity-induced cartilage degeneration [41]. The results of this present study suggest that hip OA may be affected by leptin in a similar manner; however, its association with the hip appeared stronger than that we had previously found for the knees, because the significant associations in the hips were able to be detected by traditional radiographic assessments, but those in the knees were detected only by the more-sensitive magnetic resonance imaging technique [14]. This may also reflect that radiographic JSN is a more sensitive measure for the hip than for the knee. Although no equivalent studies exist with which directly to compare ours, recent clinical studies in the knee tend to support our results. Regarding levels of leptin in knee synovial fluid, Ku *et al*. [42] reported a positive relation between leptin levels and radiographic severity of OA in a mixed group of 52 subjects, and Gandhi *et al*. [43] found that the ratio of adiponectin to leptin was negatively associated with knee pain among a mixed group of 60 subjects. In our results, it is not surprising that the association between OA and leptin decreased somewhat in magnitude after adjustment for IL-6, because leptin belongs structurally to the IL-6 family, and their serum levels were weakly related to each other in this sample.

Inflammation has been implicated in the pathogenesis of OA. Although the literature on hip synovitis is scarce, knee OA has been associated with increased levels of proinflammatory cytokines, such as IL-6, TNF (tumor necrosis factor)-α, and IL-1β in synovial fluid [44-46], and our recent study suggested that serum levels of both IL-6 and TNF-α were associated with knee cartilage loss [47]. Data from the Chingford study also reported that circulating IL-6, but not TNF-α, was associated with OA progression among women [30]. TNF-α and IL-1α can induce IL-6 production by synovial fibroblasts in the knee [48] and promote the catabolic effects of IL-6 on cartilage [49]; however, *in vitro *evidence suggests that IL-6 may reduce cartilage degeneration through inhibition of IL-1 and metalloproteinases [27,28] and promoting production of collagen II synthesis by chondrocytes [50], suggesting that IL-6 may also have beneficial effects on cartilage. Currently, no reports describe associations between hip OA severity and systemic levels of IL-6. In this study, we found that serum IL-6 was associated with hip JSN in women but not in men. This is consistent with the finding that IL-6 was cross-sectionally associated with knee ROA collapsed to 3 grades with a large sample size [30], suggesting that IL-6 also plays a detrimental role in hip OA in women. The reasons for this sex difference are unclear; however, it may reflect the influences of sex hormones in older women. In the absence of estrogen, IL-6 appears to be an upregulator of bone catabolism, leading to osteoporosis or fracture or both [31,51], although the relevance of this to hip OA is not known. Additionally, the lower sample size in the stratified analysis may have precluded the detection of a more-modest positive association between IL-6 and hip JSN in men.

Whereas mechanical loading has been suggested as an intermediary between obesity and OA in the knee and hips [52], it may not always be a contributing factor, especially in non-weight-bearing joints such as the hand [7,8]. Consistent with a recent report by Wang *et al*. [53], we found that BMI, WHR, body fat, and trunk fat were all significantly associated with hip JSN in women only; in contrast, nontrunk fat was not associated with hip JSN. This suggests that metabolic mechanisms are a possible alternative or complementary causative pathway between obesity and OA, at least for women. Indeed, we found that the positive associations between anthropometric variables and OA were largely dependent on leptin (but not IL-6), suggesting that obesity may cause cartilage damage systemically through production of leptin in adipose tissue in females. The lack of a significant association between body-fat measures and hip JSN among men may be due to modest sample size, and may also reflect a sex difference in the effect of obesity on hip OA. This is in contrast with the finding that leptin levels were significantly associated with axial JSN in men. However, leptin can be derived from nonadipose tissue, as discussed earlier, and non-fat-derived leptin can play a role in the etiology of hip OA in men. IL-6 was weakly associated with leptin and also associated with trunk and total fat ratios in our data, but did not mediate the effect of any of these fat measures on JSN. Taken together, these results suggest that leptin, rather than IL-6, is a key adipose factor involved in hip-cartilage damage. This distinction may be weaker in knee OA, in which IL-6 and leptin from the infrapatellar fat pad, with potential paracrine roles, have been shown to be expressed in greater and lesser proportions, respectively, than in thigh subcutaneous adipose tissue [54]. Any leptin-mediated effects of obesity may be complemented by a mechanical loading effect in the superior compartment, as suggested by the fact that the consistent associations between obesity measures and JSN in this compartment remained positive after adjustment for leptin. It is unknown whether mechanical loading can induce the expression of leptin within the joint, as has been shown for IL-6 [29,55]; such an effect, if any, could explain in part the leptin dependence of the associations between measures of adiposity and hip OA we report in the current study.

This study has several potential limitations. First, the modest sample size in this study could impede our discovery of less-strong associations; larger sample size is required to determine whether the insignificant results were false negative. This may explain why previous reports suggested that both leptin and IL-6 are highly expressed in osteophytes [16,29], yet we were unable to describe any associations between osteophytes and serum levels of either. Although our results are in part corroborated by published knee OA literature, we emphasize that the restriction of sample size as well as modest reliability for radiographic measures may influence the interpretation; replication of these findings within other groups of subjects is recommended.

Second, a modest response rate (57%) may have introduced selection bias into our sample. However, no significant differences were present in age, gender, and BMI between those who responded and those who did not. Whereas the sample contained subjects with some diseases, the results were largely unchanged when the analyses were adjusted for disease status or when these subjects were excluded. We measured serum total leptin levels rather than free or bound leptin levels, and the latter may have stronger associations with outcome measures; however, total leptin and free or bound leptin have similar patterns in terms of associations with body composition and gender [56].

Last, our study is of cross-sectional design, and the causative relation must be confirmed by future longitudinal studies.

## Conclusions

This cross-sectional study suggests that in older adults, serum levels of leptin in both sexes and IL-6 in women are positively associated with hip JSN. The associations between body composition and hip JSN in women are mediated by leptin. These results that suggest metabolic and inflammatory mechanisms may play roles in the etiology of hip OA, particularly in females. Replication of these findings with larger sample size is recommended.

## Abbreviations

BMI: body mass index; CV: coefficient of variation; DXA: dual energy x-ray absorptiometry; IFN-γ: interferon-γ; IL-1β: interleukin-1β; IL-1α: interleukin-1α; IL-6: interleukin-6; JSN: joint-space narrowing; MMP: matrix metalloproteinase; MRI: magnetic resonance imaging; NOS: nitric oxide synthase; OA: osteoarthritis; ROA: radiographic osteoarthritis; TASOAC: Tasmanian Older Adult Cohort; TNF-α: tumor necrosis factor α; WHR: waist-to-hip ratio.

## Competing interests

GJ serves on an advisory board and has performed clinical trials and given talks for Roche, who make an IL-6-receptor blocker. However, Roche did not fund this study nor did they have any input into the writing of this manuscript.

## Authors' contributions

GJ, FMC, and CD contributed to study design. GJ and CD contributed to the acquisition of data. OPS, GJ, FMC, and CD contributed to the analysis and interpretation of data. OPS, GJ, SJQ, FMC, DD, and CD contributed to manuscript preparation. OPS, SJQ, and CD contributed to statistical analysis.

## References

[B1] Felson DT Epidemiology of hip and knee osteoarthritis Epidemiol Rev 1988 10 1 28 3066625 10.1093/oxfordjournals.epirev.a036019

[B2] Srikanth VK Fryer JL Zhai G Winzenberg TM Hosmer D Jones G A meta-analysis of sex differences: prevalence, incidence and severity of osteoarthritis Osteoarthritis Cartilage 2005 13 769 781 10.1016/j.joca.2005.04.014 15978850

[B3] Grotle M Hagen KB Natvig B Dahl FA Kvien TK Prevalence and burden of osteoarthritis: results from a population survey in Norway J Rheumatol 2008 35 677 684 18278832

[B4] Quintana JM Arostegui I Escobar A Azkarate J Goenaga JI Lafuente I Prevalence of knee and hip osteoarthritis and the appropriateness of joint replacement in an older population Arch Intern Med 2008 168 1576 1584 10.1001/archinte.168.14.1576 18663171

[B5] Ding C Cicuttini F Scott F Cooley H Jones G Knee structural alteration and BMI: a cross-sectional study Obes Res 2005 13 350 361 10.1038/oby.2005.47 15800294

[B6] Cooper C Snow S McAlindon TE Kellingray S Stuart B Coggon D Dieppe PA Risk factors for the incidence and progression of radiographic knee osteoarthritis Arthritis Rheum 2000 43 995 1000 10.1002/1529-0131(200005)43:5<995::AID-ANR6>3.0.CO;2-1 10817551

[B7] Grotle M Hagen KB Natvig B Dahl FA Kvien TK Obesity and osteoarthritis in knee, hip and/or hand: an epidemiological study in the general population with 10 years follow-up BMC Musculoskelet Disord 2008 9 132 10.1186/1471-2474-9-132 18831740 PMC2573886

[B8] Oliveria SA Felson DT Cirillo PA Reed JI Walker AM Body weight, body mass index, and incident symptomatic osteoarthritis of the hand, hip, and knee Epidemiology 1999 10 161 166 10.1097/00001648-199903000-00013 10069252

[B9] Tepper S Hochberg MC Factors associated with hip osteoarthritis: Data from the First National Health and Nutrition Examination Survey (NHANES-I) Am J Epidemiol 1993 137 1081 1088 8317437 10.1093/oxfordjournals.aje.a116611

[B10] Franklin J Ingvarsson T Englund M Lohmander LS Sex differences in the association between body mass index and total hip or knee joint replacement resulting from osteoarthritis Ann Rheum Dis 2009 68 536 540 10.1136/ard.2007.086868 18504290

[B11] Lohmander LS De Verdier MG Rollof J Nilsson PM Engström G Incidence of severe knee and hip osteoarthritis in relation to different measures of body mass: a population-based prospective cohort study Ann Rheum Dis 2009 68 490 496 10.1136/ard.2008.089748 18467514

[B12] Lievense AM Bierma-Zeinstra SMA Verhagen AP Van Baar ME Verhaar JAN Koes BW Influence of obesity on the development of osteoarthritis of the hip: a systematic review Rheumatology (Oxford) 2002 41 1155 1162 10.1093/rheumatology/41.10.1155 12364636

[B13] Eaton CB Obesity as a risk factor for osteoarthritis: mechanical versus metabolic Med Health R I 2004 87 201 204 15354794

[B14] Ding C Parameswaran V Cicuttini F Burgess J Zhai G Quinn S Jones G Association between leptin, body composition, sex and knee cartilage morphology in older adults: the Tasmanian Older Adult Cohort (TASOAC) study Ann Rheum Dis 2008 67 1256 1261 10.1136/ard.2007.082651 18174218

[B15] Zhang Y Proenca R Maffei M Barone M Leopold L Friedman JM Positional cloning of the mouse obese gene and its human homologue Nature 1994 372 425 432 10.1038/372425a0 7984236

[B16] Dumond H Presle N Terlain B Mainard D Loeuille D Netter P Pottie P Evidence for a key role of leptin in osteoarthritis Arthritis Rheum 2003 48 3118 3129 10.1002/art.11303 14613274

[B17] Morroni M De Matteis R Palumbo C Ferretti M Villa I Rubinacci A Cinti S Marotti G In vivo leptin expression in cartilage and bone cells of growing rats and adult humans J Anat 2004 205 291 296 10.1111/j.0021-8782.2004.00333.x 15447688 PMC1571344

[B18] Loeser RF Systemic and local regulation of articular cartilage metabolism: where does leptin fit in the puzzle? Arthritis Rheum 2003 48 3009 3012 10.1002/art.11315 14613260

[B19] Härle P Straub RH Leptin is a link between adipose tissue and inflammation Ann N Y Acad Sci 2006 1069 454 462 10.1196/annals.1351.044 16855173

[B20] Baumann H Morella KK White DW Dembski M Bailon PS Kim H Lai CF Tartaglia LA The full-length leptin receptor has signaling capabilities of interleukin 6-type cytokine receptors Proc Natl Acad Sci USA 1996 93 8374 8378 10.1073/pnas.93.16.8374 8710878 PMC38678

[B21] Zhang F Basinski MB Beals JM Briggs SL Churgay LM Clawson DK DiMarchi RD Furman TC Hale JE Hsiung HM Schoner BE Smith DP Zhang XY Wery JP Schevitz RW Crystal structure of the obese protein leptin-E100 Nature 1997 387 206 209 10.1038/387206a0 9144295

[B22] Pottie P Presle N Terlain B Netter P Mainard D Berenbaum F Obesity and osteoarthritis: more complex than predicted! Ann Rheum Dis 2006 65 1403 1405 10.1136/ard.2006.061994 17038451 PMC1798356

[B23] Simopoulou T Malizos KN Iliopoulos D Stefanou N Papatheodorou L Ioannou M Tsezou A Differential expression of leptin and leptin's receptor isoform (Ob-Rb) mRNA between advanced and minimally affected osteoarthritic cartilage; effect on cartilage metabolism Osteoarthritis Cartilage 2007 15 872 883 10.1016/j.joca.2007.01.018 17350295

[B24] Goldring MB The role of the chondrocyte in osteoarthritis Arthritis Rheum 2000 43 1916 1926 10.1002/1529-0131(200009)43:9<1916::AID-ANR2>3.0.CO;2-I 11014341

[B25] Sakao K Takahashi KA Mazda O Arai Y Tonomura H Inoue A Saito M Fujioka M Takamiya H Imanishi J Kubo T Enhanced expression of interleukin-6, matrix metalloproteinase-13, and receptor activator of NF-κB ligand in cells derived from osteoarthritic subchondral bone J Orthop Sci 2008 13 202 210 10.1007/s00776-008-1227-5 18528653

[B26] Mohamed-Ali V Goodrick S Rawesh A Katz DR Miles JM Yudkin JS Klein S Coppack SW Subcutaneous adipose tissue releases interleukin-6, but not tumor necrosis factor-α, in vivo J Clin Endocrinol Metab 1997 82 4196 4200 10.1210/jc.82.12.4196 9398739

[B27] Palmer G Guerne PA Mezin F Maret M Guicheux J Goldring MB Gabay C Production of interleukin-1 receptor antagonist by human articular chondrocytes Arthritis Res 2002 4 226 231 10.1186/ar411 12010575 PMC111027

[B28] Silacci P Dayer JM Desgeorges A Peter R Manueddu C Guernet PA Interleukin (IL)-6 and its soluble receptor induce TIMP-1 expression in synoviocytes and chondrocytes, and block IL-1-induced collagenolytic activity J Biol Chem 1998 273 13625 13629 10.1074/jbc.273.22.13625 9593700

[B29] Sakao K Takahashi KA Arai Y Saito M Honjo K Hiraoka N Asada H Shin-Ya M Imanishi J Mazda O Kubo T Osteoblasts derived from osteophytes produce interleukin-6, interleukin-8, and matrix metalloproteinase-13 in osteoarthritis J Bone Miner Metab 2009 27 412 423 10.1007/s00774-009-0058-6 19333684

[B30] Livshits G Zhai G Hart DJ Kato BS Wang H Williams FMK Spector TD Interleukin-6 is a significant predictor of radiographic knee osteoarthritis: the Chingford Study Arthritis Rheum 2009 60 2037 2045 10.1002/art.24598 19565477 PMC2841820

[B31] Ding C Parameswaran V Udayan R Burgess J Jones G Circulating levels of inflammatory markers predict change in bone mineral density and resorption in older adults: a longitudinal study J Clin Endocrinol Metab 2008 93 1952 1958 10.1210/jc.2007-2325 18285417

[B32] Altman RD Hochberg M Murphy WAJ Wolfe F Lequesne M Atlas of individual radiographic features in osteoarthritis Osteoarthritis Cartilage 1995 3 3 70 8581752

[B33] Zhai G Cicuttini F Srikanth V Cooley H Ding C Jones G Factors associated with hip cartilage volume measured by magnetic resonance imaging: the Tasmanian Older Adult Cohort Study Arthritis Rheum 2005 52 1069 1076 10.1002/art.20964 15818695

[B34] Williams R Generalized ordered logit/partial proportional odds models for ordinal dependent variables Stata J 2006 6 58 82

[B35] Cornish J Callon KE Bava U Lin C Naot D Hill BL Grey AB Broom N Myers DE Nicholson GC Reid IR Leptin directly regulates bone cell function in vitro and reduces bone fragility in vivo J Endocrinol 2002 175 405 415 10.1677/joe.0.1750405 12429038

[B36] Maor G Rochwerger M Segev Y Phillip M Leptin acts as a growth factor on the chondrocytes of skeletal growth centers J Bone Miner Res 2002 17 1034 1043 10.1359/jbmr.2002.17.6.1034 12054158

[B37] Figenschau Y Knutsen G Shahazeydi S Johansen O Sveinbjörnsson B Human articular chondrocytes express functional leptin receptors Biochem Biophys Res Commun 2001 287 190 197 10.1006/bbrc.2001.5543 11549273

[B38] Otero M Lago R Lago F Reino JJ Gualillo O Signalling pathway involved in nitric oxide synthase type II activation in chondrocytes: synergistic effect of leptin with interleukin-1 Arthritis Res Ther 2005 7 R581 R591 10.1186/ar1708 15899045 PMC1174950

[B39] Otero M Gomez Reino JJ Gualillo O Synergistic induction of nitric oxide synthase type II: in vitro effect of leptin and interferon-γ in human chondrocytes and ATDC5 chondrogenic cells Arthritis Rheum 2003 48 404 409 10.1002/art.10811 12571850

[B40] Presle N Pottie P Dumond H Guillaume C Lapicque F Pallu S Mainard D Netter P Terlain B Differential distribution of adipokines between serum and synovial fluid in patients with osteoarthritis: contribution of joint tissues to their articular production Osteoarthritis Cartilage 2006 14 690 695 10.1016/j.joca.2006.01.009 16527497

[B41] Griffin TM Huebner JL Kraus VB Guilak F Extreme obesity due to impaired leptin signaling in mice does not cause knee osteoarthritis Arthritis Rheum 2009 60 2935 2944 10.1002/art.24854 19790050 PMC2829313

[B42] Ku JH Lee CK Joo BS An BM Choi SH Wang TH Cho HL Correlation of synovial fluid leptin concentrations with the severity of osteoarthritis Clin Rheumatol 2009 28 1431 1435 10.1007/s10067-009-1242-8 19662330

[B43] Gandhi R Takahashi M Smith H Rizek R Mahomed NN The synovial fluid adiponectin-leptin ratio predicts pain with knee osteoarthritis Clin Rheumatol 2010 in press 10.1007/s10067-010-1429-z 20349258

[B44] Partsch G Steiner G Leeb BF Dunky A Bröll H Smolen JS Highly increased levels of tumor necrosis factor-α and other proinflammatory cytokines in psoriatric arthritis synovial fluid J Rheumatol 1997 24 518 523 9058659

[B45] Smith MD Triantafillou S Parker A Youssef PP Coleman M Synovial membrane inflammation and cytokine production in patients with early osteoarthritis J Rheumatol 1997 24 365 371 9034998

[B46] Vignon E Balblanc JC Mathieu P Louisot P Richard M Metalloprotease activity, phospholipase A2 activity and cytokine concentration in osteoarthritis synovial fluids Osteoarthritis Cartilage 1993 1 115 120 10.1016/S1063-4584(05)80026-3 8886087

[B47] Ding C Serum levels of inflammatory markers, knee radiographic osteoarthritis, and knee cartilage loss in older adults J Intern Med 2009 39 A47

[B48] Bondeson J Wainwright SD Lauder S Amos N Hughes CE The role of synovial macrophages and macrophage-produced cytokines in driving aggrecanases, matrix metalloproteinases, and other destructive and inflammatory responses in osteoarthritis Arthritis Res Ther 2006 8 R187 10.1186/ar2099 17177994 PMC1794533

[B49] Flannery CR Little CB Hughes CE Curtis CL Caterson B Jones SA IL-6 and its soluble receptor augment aggrecanase-mediated proteoglycan catabolism in articular cartilage Matrix Biol 2000 19 549 553 10.1016/S0945-053X(00)00111-6 11068209

[B50] Namba A Aida Y Suzuki N Watanabe Y Kawato T Motohashi M Maeno M Matsumura H Matsumoto M Effects of IL-6 and soluble IL-6 receptor on the expression of cartilage matrix proteins in human chondrocytes Connect Tissue Res 2007 48 263 270 10.1080/03008200701587513 17882702

[B51] Jilka RL Hangoc G Girasole G Passeri G Williams DC Abrams JS Boyce B Broxmeyer H Manolagas SC Increased osteoclast development after estrogen loss: Mediation by interleukin-6 Science 1992 257 88 91 10.1126/science.1621100 1621100

[B52] Griffin TM Guilak F The role of mechanical loading in the onset and progression of osteoarthritis Exerc Sport Sci Rev 2005 33 195 200 10.1097/00003677-200510000-00008 16239837

[B53] Wang Y Simpson JA Wluka AE Teichtahl AJ English DR Giles GG Graves S Cicuttini FM Relationship between body adiposity measures and risk of primary knee and hip replacement for osteoarthritis: a prospective cohort study Arthritis Res Ther 2009 11 R31 10.1186/ar2636 19265513 PMC2688176

[B54] Distel E Cadoudal T Durant S Poignard A Chevalier X Benelli C The infrapatellar fat pad in knee osteoarthritis: an important source of interleukin-6 and its soluble receptor Arthritis Rheum 2009 60 3374 3377 10.1002/art.24881 19877065

[B55] Sanchez C Gabay O Salvat C Henrotin YE Berenbaum F Mechanical loading highly increases IL-6 production and decreases OPG expression by osteoblasts Osteoarthritis Cartilage 2009 17 473 481 10.1016/j.joca.2008.09.007 18974013

[B56] McConway MG Johnson D Kelly A Griffin D Smith J Wallace AM Differences in circulating concentrations of total, free and bound leptin relate to gender and body composition in adult humans Ann Clin Biochem 2000 37 717 723 10.1258/0004563001899771 11026527

